# Recurrent intussusception in a child revealing a neuroendocrine tumor within a Meckel's diverticulum: a case report

**DOI:** 10.1093/jscr/rjaf085

**Published:** 2025-02-28

**Authors:** Hussein Naji, Reem Allateef, Aftab Ahmed, Shahzad Yousaf, Ghassan Nakib, Rawan Alhalabi

**Affiliations:** Pediatric Surgery Department, Mediclinic Parkview Hospital, Albarsha, Dubai, UAE; Family Medicine Department, Mediclinic Parkview Hospital, Albarsha, Dubai, UAE; Pediatric Surgery Department, Mediclinic Parkview Hospital, Albarsha, Dubai, UAE; General Surgery Department, Mediclinic Parkview Hospital, Albarsha, Dubai, UAE; Pediatric Surgery Department, Mediclinic Parkview Hospital, Albarsha, Dubai, UAE; Pediatric Department, American Hospital Dubai, Oud Metha, Dubai, UAE

**Keywords:** Meckel's diverticulum, intussusception, neuroendocrine tumor (NET), acute abdomen, small intestine

## Abstract

Meckel's diverticulum (MD) is a common congenital anomaly of the gastrointestinal tract, often asymptomatic but occasionally presenting with various complications. Neuroendocrine tumors (NETs) are slow-growing neoplasms that mostly originate from the small intestine. The typical age for the presence of a carcinoid tumor within MD is above 50s. NETs reported to be the most common primary malignancy originating from MD in elderly. We report the first case of a female child who demonstrated five episodes of intussusception, ending in surgical resection revealing a NET within MD. The histopathology study proved a low-grade tumor with no evidence of necrosis. Current literature lacks definitive guidelines for managing NET in MD cases, especially in pediatric populations, necessitating further research for optimal treatment strategies. Prompt removal of the diverticulum upon discovery is advised to enable early detection and treatment of carcinoid tumors, thereby avoiding potential complications or advanced disease stages.

## Introduction

The omphalomesenteric duct is a canal that connects the midgut to the yolk sac in the fetus. Ideally involutes during the 5th-8th weeks of gestation, providing nutritional support until the formation of the placenta [1]. Meckel's diverticulum (MD) is an embryonic remnant pouch resulting from incomplete obliteration of the omphalomesenteric duct [1–3]. It is generally asymptomatic but may present with painless rectal bleeding, intussusception, diverticulitis, axial torsion, perforation, or peritonitis [1–3]. It is known to contain small intestinal tissue and gastric mucosa and has been documented in adults only to harbour tumors [1–4]. In this report, we present a novel case of recurrent intussusception in a child, which after surgical resection revealed a neuroendocrine tumor (NET) within a MD.

## Case report

**Figure 1:**
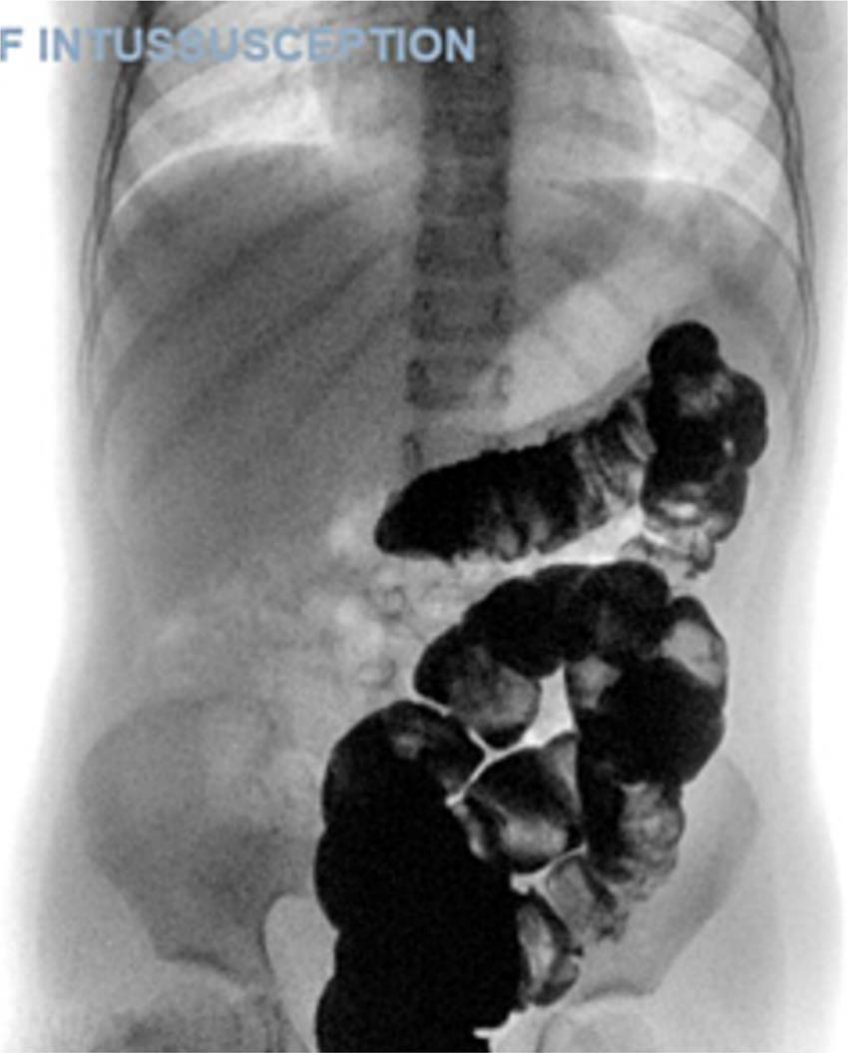
Therapeutic enema for intussusception reduction (3rd attack).

A 5-year-old female presented with a 1-day history of abdominal pain and vomiting. Physical examination revealed abdominal tenderness on the right side. The patient had a history of four previous episodes of intussusception, all of which were conservatively reduced with hydrostatic contrast enema (Fig. 1). The most recent episode occurred 3 months prior to the current presentation. An MRI enterography performed after the fourth episode showed no abnormalities.

**Figure 2:**
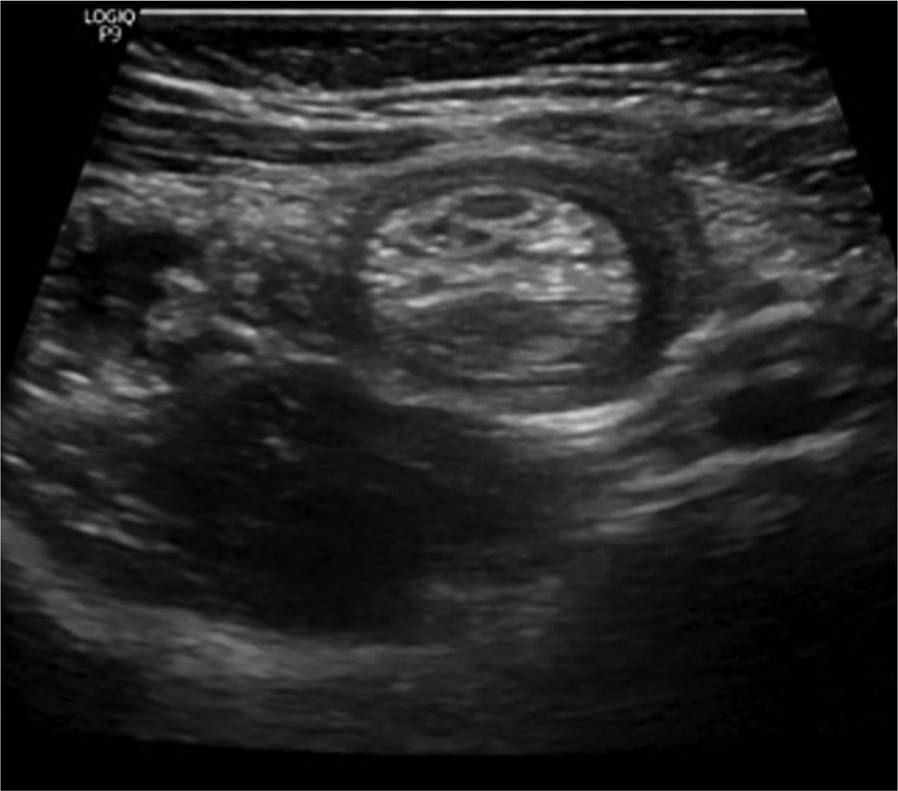
Ultrasound of the abdomen showed a target sign of intussusception (5th attack).

**Figure 3:**
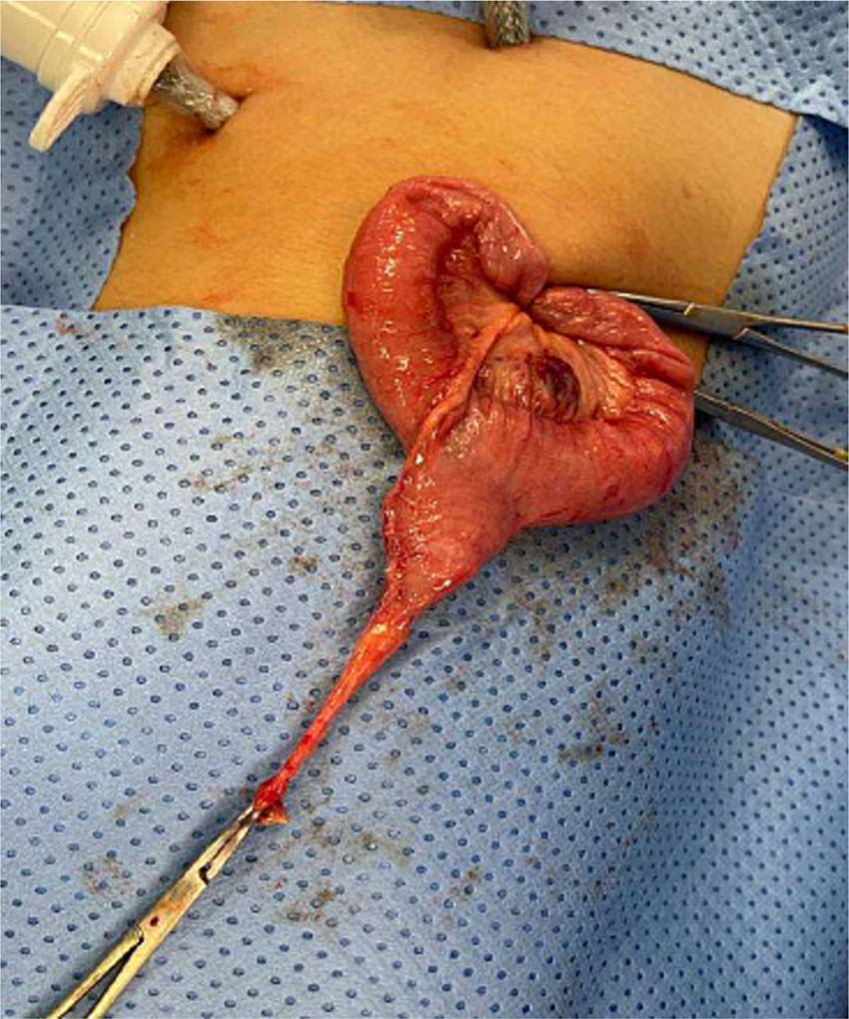
Meckel’s diverticulum as a lead point for recurrent intussusception.

The blood tests showed raised inflammatory markers. An abdominal ultrasound confirmed the presence of intussusception (Fig. 2). A decision was made for a laparoscopic reduction of the intussusception and inspecting for a leading point. A MD was demonstrated and resected accordingly (Fig. 3). The location of intussusception was at the ileo-cecal area. The affected bowel was viable. The position of the MD was in the terminal ileum and it was the lead point for the intussusception. No lymph node involvement. Also, a tumor located at the base of the MD ~4 mm from the resection margin, was found and resected.

**Figure 4:**
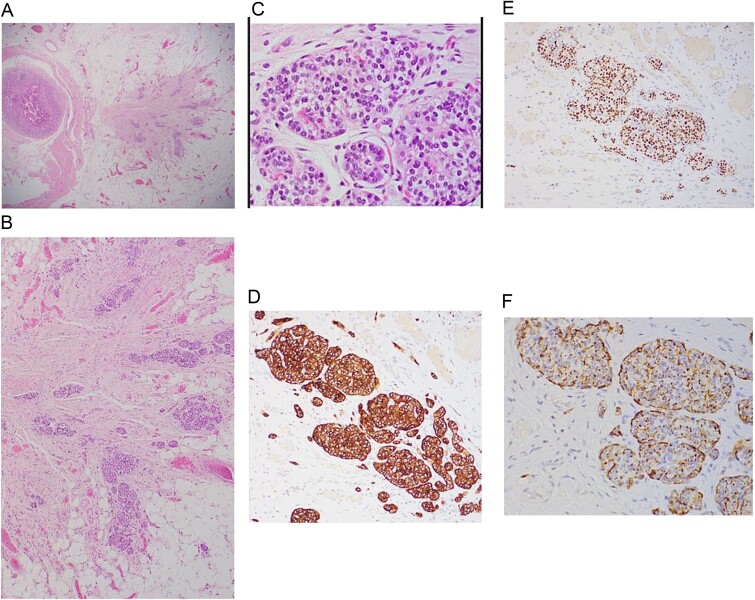
A: Low power magnification of H&E stain; tumor nest in the wall of the bowel, ribbons and cords of monomorphic cells with salt-pepper chromatin pattern, typically seen in endocrine tumors. B: Neuroendocrine tumor in the wall of bowel and fat (×20 magnification). C: Solid nest of tumor (high power magnification ×40). D: Immunohistochemistry Synaptophysin positive; Synaptophysin stains vesicles in neuroendocrine cells. E: Immunohistochemistry INSMI positive, a specific marker for neuroendocrine. F: Immunohistochemistry AE1/AE3 positive, a neuroendocrine marker.

Histopathological assessment (Fig. 4) revealed small intestinal mucosa showing ectopic gastric tissue composed of foveolar and oxynetic glands, compatible with MD. In addition, a tumor was found. Composed of ribbons and cords of monomorphic cells with salt-pepper chromatin pattern and plenty of cytoplasm that show strong expression of Synaptophysin, INSMI, and Chromogranin A. Ki-67 proliferation index is low; <1% of tumor cells, compatible with NET grade 1. The tumor measured 4 mm, with no evidence of perineural or vascular invasion. Following the surgery, the child made a complete recovery and remained free of further episodes of intussusception during one year of follow-up in the clinic.

## Discussion

MD is usually lined by two types of mucosae: the innate intestinal mucosa and a heterotopic mucosa, with the gastric mucosa being a common heterotopic variant [5, 6]. Roughly, <15% of patients might exhibit extra tissue within the diverticulum, and the incidence of containing a primary tumor is as low as 0.5%, only reported in adults in the literature [1–5].

Carcinoid tumors, recently known as NETs, are slow-growing neoplasms arising from enterochromaffin cells with the ability to secrete a diversity of proteins and hormones. Most commonly originates from the small intestine, primarily the ileum [5–7].

Generally, ~80% of the affected people are symptomless. The known presentations are non-specific. Consequently, 50% of the patients will be misdiagnosed for at least 2–7 years, resulting in advanced stages of malignancy with up to 70% demonstrating metastasis [5–8].

The typical age for the presence of a carcinoid tumor within MD is 55 years, with a double occurrence in men compared to women [6, 8]. Modlin *et al*. stated that less than 1% of all carcinoids in adults appear in the MD [8].

Two papers published involved children. However, neither paper involved an incidental diagnosis of NET. The first article, published in 1969 [9, 10], included 40 cases, with only a patient of 12 years old with diverticulitis, not recurrent intussusceptions.

The second article, published in 2004, is an analysis of 13 715 cases of carcinoid tumors from 1950 to 1999. A 14-month-old child with hematochezia, reported in 1996 [8]. These studies illustrate the rarity of neuroendocrine tumors in MD, especially in children. Our case, which uniquely combines recurrent intussusceptions with a NET in MD, has not been previously published.

The Surveillance, Epidemiology, and End Results Program of the National Cancer Institute reported NETs as the most common primary malignancy originating from MD (76.5%), followed by adenocarcinoma (11.4%), GI stromal tumor (10.8%), and lymphoma (1.3%) [4–6].

The WHO issued a classification that categorizes these tumors as Grade 1 (G1-NET), Grade 2 (G2-NET), Grade 3 - neuroendocrine carcinoma (G3-NEC) large- or small-cell type, and mixed adenoneuroendocrine carcinoma. However, this grading is based on mitotic count besides using Ki-67 index. G1- NET is known to be composed of tumor cells dominating round or oval nuclei with salt-pepper chromatin and eosinophilic granular cytoplasm. G1-NET is concluded by a mitotic count of <2 per 2 mm^2^ (10 high power fields [HPF], 40× magnification) and/or ≤ 2% Ki-67 index. Mostly, the used stains are synaptophysin, chromogranin, CDX2 (identifying the origin, mainly reflects the intestine), PAX8 (positive in the majority of rectal and pancreatic), CD56 / NCAM1 (low specificity) and CEA [8–13].

In our case, synaptophysin, chromogranin A, and INSMI stains were positive, and Ki-67 index is <1%, leading to the diagnoses of G1-NET.

In adults, the treatment of MD-related NET typically follows staging guidelines. Regular exclusively MD surgical removal is considered satisfactory for lesions <10 mm [14–16], while substantial tumors may require resection of the ileal tract and corresponding mesentery. Metastatic lesions are managed with chemotherapy and symptomatic inhibition therapy [4, 17].

Although most literature supports surgical resection of incidental MD [18–20], the association with NETs remains rare, leading to inconclusive findings in reported studies in adults. Existing publications are lacking evidence-based guidelines, being absent particularly in pediatric cases.

Based on our experience, we recommend performing exploratory laparoscopy after the third recurrence of intussusceptions in children above 2 years old. Excision of the diverticulum upon identification is recommended to facilitate early recognition and management of carcinoid tumors, thereby preventing potential sequelae, or presenting with advanced stages.

MD remains a clinically significant entity, often associated with rare but potentially malignant complications such as NET. Timely recognition and surgical intervention are fundamental for optimal management and prevention of adverse outcomes in affected individuals.
